# A Population Survival Kinetics Assessment of Extensive Small Cell Lung Cancer and Rationale for Maintenance Therapy

**DOI:** 10.3390/curroncol32050258

**Published:** 2025-04-29

**Authors:** David J. Stewart, Katherine Cole, Stephanie Brule

**Affiliations:** Faculty of Medicine, Department of Medicine, Division of Medical Oncology, University of Ottawa, 501 Smyth Road, Ottawa, ON K1H 8L6, Canada; katcole@toh.ca (K.C.); sbrule@toh.ca (S.B.)

**Keywords:** population survival kinetics, small cell lung cancer, chemotherapy, PD-1/PD-L1 inhibitors, overall survival, progression-free survival

## Abstract

Progression-free survival (PFS) and overall survival (OS) curves generally approximate first-order kinetics. On log-linear plots, convex curves with downward inflection (indicating late acceleration of progression/death) might arise from stopping effective therapies. We digitized published PFS/OS curves for etoposide/platinum-treated extensive small-cell lung cancer (SCLC) and other malignancies and replotted the curves log-linearly. Of 26 SCLC PFS curves, 21 (81%) were highly convex (with a marked late down-turn), and 26 (100%) were moderately or highly convex vs. 35/888 (4%) highly convex and 186 (21%) moderately/highly convex curves for other cancers (*p* < 0.0001). For SCLC, all 32 OS curves were moderately or highly convex vs. 87/363 (24%) that were moderately/highly convex for other cancers (*p* < 0.0001). The SCLC PFS curves had an initial downward inflection at a median of 3.1 months (around the completion of first-line chemotherapy), then a second inflection at 5.4 months, with further acceleration of progression. The median PFS half-life was 11.9 months while receiving treatment vs. 1.7 months after the second inflection point. Immunotherapy benefit appeared to be limited to 6–10% of the population. SCLC PFS/OS curves are more often convex than for other cancers, reflecting SCLC chemotherapy sensitivity but rapid progression following the completion of first-line chemotherapy. Effective maintenance strategies are needed.

## 1. Introduction

Small-cell lung cancer (SCLC) grows rapidly. Without chemotherapy, median life expectancy is about 6 weeks for patients with extensive stage disease (i.e., patients with stage M1 disease) and 3 months for patients with limited stage disease (i.e., patients with stage M0 disease) [1]. However, SCLC is also sensitive to chemotherapy, with rapid clinical improvement and high response rates of 50–85% in the first-line setting [2]. The median life expectancy for extensive SCLC patients treated with chemotherapy is 8–12 months [2]. The combination of a platinum (cisplatin or carboplatin) with etoposide is the most commonly used regimen for SCLC, although other regimens such as irinotecan–cisplatin and cyclophosphamide/doxorubicin/vincristine (CAV) have similar activity [2].

Typically, 4–6 cycles of first-line chemotherapy are given, followed by a break, with the option of restarting chemotherapy upon tumor regrowth [2]. Some trials have suggested an OS benefit from increasing the number of cycles of first-line chemotherapy [3,4], while others have shown only a modest impact on PFS and not OS [5] or an OS impact only in patients achieving complete remission during initial therapy [6]. However, recent studies have demonstrated improved OS with the addition of a PD-1/PD-L1 inhibitor to chemotherapy [7,8].

Many biological processes follow first-order kinetics. For processes that follow first-order kinetics, in a given period of time, a given proportion rather than a given amount of the remaining factor of interest disappear per unit time. Some examples of first-order kinetics include drug disappearance from blood (as a basis for pharmacokinetics) and radioactive decay. Most PFS and OS curves can also be fit by first-order kinetic models, and assessments of PFS and OS first-order kinetics are examples of “population survival kinetics” [9,10,11,12,13].

When curves for processes that follow first-order kinetics are replotted on a log-linear scale (log % remaining vs. linear time), they convert from a curved line to a straight line. Plotting survival on a log-linear scale can have several advantages over Kaplan–Meier plots [14], and deviations of first-order kinetic log-linear plots from a straight line can give clinical and biological insights [11,12,14]. For example, in pharmacokinetics, the log-linear curve is initially straight as the drug is absorbed into tissues, but, when the tissues become saturated, the curve bends to the right, showing “2-phase decay” as the slower processes of metabolism and excretion become the predominant mechanisms for further drug disappearance. Pharmacokinetics use mathematical modeling methods such as exponential decay nonlinear regression analysis (EDNLRA) of the drug disappearance curve to gain insight into the biology of drug disappearance.

**Figure 1:**
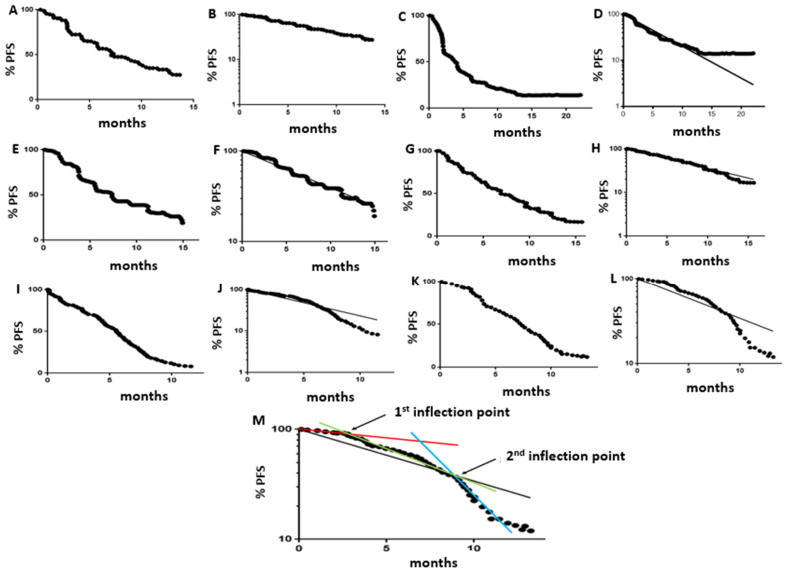
(**A**) Linear-linear plot of a 1-phase decay PFS curve. (**B**) Log-linear plot of the same 1-phase decay curve, with the PFS curve lying along the 1-phase decay model regression line. (**C**) Linear-linear plot of a 2-phase decay PFS curve. (**D**) Log-linear plot of a 2-phase decay PFS curve, with the PFS curve initially lying along or to the left of the 1-phase decay model regression line, then deviating to the right across the regression line. (**E**) Linear-linear plot of what we classified as a “variable 1-phase” PFS curve. (**F**) Log-linear plot of a “variable 1-phase” PFS curve, with an irregular or undulating PFS curve that has the same overall slope as the 1-phase decay regression model and is functionally similar to a 1-phase decay curve. We referred to this curve shape as “S-shaped” in earlier publications [10]. (**G**) Linear-linear plot of a low-convexity PFS curve. (**H**) Log-linear plot of this low-convexity PFS curve that initially follows the 1-phase decay model regression line, with a late minor downward inflection, functionally similar to a 1-phase decay curve. (**I**) Linear-linear plot of a moderate convexity PFS curve. (**J**) Log-linear plot of this moderate-convexity PFS curve that initially is slightly above the 1-phase decay model regression line, then deviates downward across it. (**K**) Linear-linear plot of a high-convexity PFS curve. (**L**) Log-linear plot of this high-convexity PFS curve that is initially substantially above the 1-phase decay model regression line, then has a substantial downward deviation (functionally similar to a moderate-convexity curve). (**M**) Log-linear high-convexity curve showing what we characterized as first and second downward inflection points, with increasingly rapid progression. The red line is the manually constructed “best fit” for the initial part of the curve, and the blue line is the “best fit” line for the terminal portion of the curve. The first inflection point is the estimated point at which the log-linear PFS curve deviates downward from the red best-fit line, and the second inflection point is the estimated point at which the log-linear PFS curve meets the blue best-fit line. The green line is the manually constructed best-fit line between the 2 inflection points.

In PFS/OS population survival kinetics, an inflection point with the deviation of a log-linear curve to the right (i.e., two-phase decay) may indicate two distinct subpopulations with differing rates of progression or death [12,13]. Conversely, a downward inflection that produces log-linear curve convexity suggests late acceleration of progression or death due to factors such as stopping effective therapy or the accelerated aging of a population [11,12,14]. Curve convexity might identify situations where one should explore new maintenance strategies to reduce the acceleration of tumor growth after the completion of first-line therapy. Examples of log-linear PFS curves that show one-phase decay vs. two-phase decay vs. varying degrees of convexity are presented in Figure 1.

The log-linear curve shape varies significantly with tumor and therapy type, and we hypothesized that this arises from both biological factors and from usual clinical practices [9,10,11,12,13]. These earlier analyses suggested that PFS and OS curves for extensive SCLC are particularly likely to be highly convex, indicating the acceleration of progression and death with increasing time from therapy initiation [12]. In this paper, we more fully assess the characteristics of extensive SCLC PFS and OS curves as well as compare them to the characteristics of curves we previously reported for multiple other tumor and therapy types [9,10,12]. In addition, for patients undergoing treatment for extensive SCLC, the optimal frequency of follow-up scans is uncertain, although it is typically every 6–9 weeks. We used these analyses to further assess optimal scan frequency.

## 2. Methods

We searched PubMed until November 2019, restricted to English language publications, to identify published studies on the use of a platinum (cisplatin or carboplatin) combined with etoposide in extensive SCLC. PubMed search terms included “cisplatin OR carboplatin”, “etoposide”, “small cell NOT non small cell”, “lung”, “progression-free survival AND overall survival”, and “extensive”, with the filter “clinical trial”. We excluded studies that included limited SCLC unless separate data were reported for the extensive SCLC subgroup. We also excluded studies that did not have published PFS or OS curves; studies in which additional chemotherapy agents were given concurrently, sequentially or alternating with platinum–etoposide; and studies for which the published PFS or OS curves were generated using fewer than 50 patients. The restriction to PFS or OS curves generated using a minimum of 50 or more patients was arbitrary, based on the assumption that higher patient numbers would result in more reproducible data, but we did not conduct analyses based on curves derived from fewer patients to test this assumption. Studies adding a targeted therapy or immunotherapy to platinum–etoposide were included if other criteria were met.

Published PFS and OS curves were digitized using an online application (https://apps.automeris.io/wpd/) (accessed 28 April 2025). GraphPad Prism 7 (GraphPad Software, La Jolla, CA, USA) was then used for EDNLRA of the digitized data to generate 1-phase exponential decay models, as previously described [9,10,12,13,15]. Also as previously described [9,10,12], we excluded data from the potentially highly variable terminal portion of the curves estimated to have fewer than 10 remaining patients. Including the terminal portion of the curves would not have changed any of the conclusions in this manuscript.

Constraints were set at Y_o_ = 100% (since PFS and OS started at 100%) and plateau = 0% (since all patients would eventually progress or die if followed long enough). Curve fitting was generally substantially better with vs. without these constraints. These analyses permitted the production of log-linear plots and the estimation of PFS and OS half-lives (time to progression or death of one-half of the remaining patients). PFS and OS half-lives are very similar to and correlate strongly with medians [9] but offer advantages over the use of medians because of some of the calculations they may facilitate [9,10,11,12,13,15]. For example, use of half-lives makes PFS gain a better predictor of OS gain in a trial [9] and facilitates the calculation of the optimal frequency of follow-up scans for patients on therapy [10], the time course of recurrence after potentially curative therapy [13], and the proportion of patients who die per week that initiation of therapy is delayed [15].

Based on log-linear shape, each PFS and OS curve was then classified as fitting 1 of 6 previously defined [9,10] curve shapes (Figure 1A–L): 1-phase (approximating a straight line on log-linear plots, along the 1-phase decay model regression line), 2-phase (along or to left of the 1-phase decay model regression line in the early part of curve, with deviation to the right of the 1-phase decay curve in the later part of the curve), variable 1-phase (irregular or undulating but with an overall slope matching the 1-phase decay model regression line, functionally equivalent to 1-phase curves [called “S-shaped” in some of our earlier publications [10]]), low convexity (matching the 1-phase decay model regression line initially, with a minor downturn at the end of the curve, functionally equivalent to 1-phase curves), moderately convex (modest deviation above the 1-phase decay model regression line in the early part of the curve and modest deviation below the 1-phase decay model regression line towards the end of the curve), or highly convex (substantial deviation above the 1-phase decay model regression line in the early part of the curve, then substantial deviation below the 1-phase decay model regression line in the later part of the curve) (Figure 1).

The SCLC PFS and OS curves were compared to those we previously reported for multiple other tumor and therapy types [9,10,12] with respect to the proportion of the curves that were moderately or highly convex.

Most SCLC PFS and OS curves appeared upon inspection to have an early downward inflection point, with acceleration of progression or death, and a later second inflection point, with further acceleration of progression or death. To estimate the time of these inflection points, we manually created a “best fit” line along the early part of the curve and another “best fit” line along the terminal portion of the curve. The first inflection point was the point at which the PFS or OS log-linear curve moved downward from the initial “best fit” line, and the second inflection point was the point at which the PFS or OS log-linear curve began to match the terminal “best fit” line. Figure 1M presents an example of a highly convex PFS curve, with an illustration of “best fit” lines and identification of the first and second inflection points.

Some patients who experience tumor progression following the discontinuation of first-line chemotherapy may benefit from subsequent treatment, but there is no consensus on how frequently one should perform follow-up scans on patients with SCLC who have completed first-line chemotherapy. To assess this, we calculated the PFS half-life on the terminal portion of the PFS curves following therapy discontinuation, then calculated the proportion of remaining patients who would have progressed by the next scan for scans performed at different time intervals, as previously described [10]. For this, we used the EXCEL (Microsoft, Redmond, WA, USA) formula:

X = EXP(−t_n_∗0.693/t_1/2_) (1)

where X is the proportion remaining progression-free at the time interval of interest, EXP signifies exponential, t_n_ is the time interval of interest between scans (e.g., 3 weeks, 6 weeks, etc.), ∗ indicates multiplication, 0.693 is the natural log of 2, and t_1/2_ is the PFS half-life in weeks.

In using this formula, we made the assumption that PFS generally approximates first-order kinetics, in keeping with our findings across multiple tumor types and therapies in earlier assessments [9,10,11,12,13,15]. The percentage of remaining patients who would have progressed since the last scan = 100 − (X∗100).

We also calculated the PFS half-life after the second inflection point on convex curves, the proportion of patients who would progress between scans performed at different intervals following this second PFS curve inflection point, and the 95% confidence intervals and R^2^ metrics.

Since our earlier analyses suggested that immune checkpoint inhibitors are generally associated with PFS curves that exhibit 2-phase decay (with an inflection point to the right) [12], we did additional landmark analyses for the time after the completion of first-line chemotherapy for the two trials involving PD-1/PD-L1 immune checkpoint inhibitors [7,8]. We also added analyses from a third trial [16] that was published after the 2019 data cutoff date for the primary analyses.

For these analyses, we used published methods [12] to perform 2-phase exponential decay nonlinear regression analyses of PFS curves starting at 3 weeks after initiation of the final course of induction chemotherapy. For these analyses, we used the entire residual portion of the PFS curve rather than truncating curves at the point where there were fewer than 10 remaining patients. This identified 2 distinct subpopulations (one with rapid progression and one with slow progression) and permitted the calculation of the relative size and PFS half-life for each subpopulation.

## 3. Results

### 3.1. Studies Included

A total of 26 SCLC PFS curves and 32 SCLC OS curves met our criteria of being derived only from patients with extensive disease, including data from 50 or more patients, and with no concurrent, alternating, or subsequent first-line chemotherapy agents other than a platinum and etoposide [7,8,17,18,19,20,21,22,23,24,25,26,27,28,29,30,31,32,33,34]. As noted, while studies including other chemotherapy agents were excluded, we did include studies adding targeted agents or immunotherapy to platinum–etoposide. We did not track the number of publications excluded based on not meeting our eligibility criteria. Table 1 presents the study characteristics and median PFS and OS.

### 3.2. Population Kinetics Characteristics of PFS Curves

Table 2 includes the EDNLRA characteristics of the PFS curves, including curve shape on log-linear plots (as illustrated in Figure 1), PFS half-life, 95% confidence intervals, R^2^ values, and related calculations. We previously reported that PFS and OS half-lives generally correlate strongly with medians [9], and, in this analysis, the median value for the PFS half-lives across the studies was the same as the median value for the PFS medians across studies, at 5.4 months. The first downward inflection point in the PFS curve occurred at a median of 3.1 months, around the end of the planned first-line therapy, with a median of 82% of patients still progression-free at the time of the first downward inflection point.

The second downward inflection point (with further acceleration of progression) was noted at a median of 5.4 months after therapy initiation and 2.3 months after the first downward inflection point, with 60% of patients still progression-free at the time of the second inflection point. The PFS half-life was a median of 11.9 months while patients were still on therapy vs. 2.6 months after completion of first-line chemotherapy and 1.7 months after the second downward inflection point on the PFS curve.

### 3.3. Optimal Frequency of Follow-Up Scans

Table 3 lists the proportion of remaining progression-free patients who would have progressed by the next scan for scans conducted at different time intervals. While receiving first-line chemotherapy, only 8% of patients would have progressed by the first follow-up scan if conducted at 6 weeks, and 15% would have progressed by the next scan if it were only performed at 12 weeks. For the 60% of patients who made it to the second PFS curve downward inflection point without progressing, 24% would already have progressed by the next scan if conducted 3 weeks later and 43% if the next scan was conducted 6 weeks later.

### 3.4. Population Kinetics Characteristics of Overall Survival Curves

Table 4 includes the EDNLRA characteristics of the OS curves. The median value for the OS half-life across the studies was 10.1 months (compared to 10.2 months for the median OS). The first downward inflection point in the OS curve occurred at a median of 5.5 months (about 1.7 months after the end of planned first-line therapy), with a median of 83% of patients still alive at the time of the first downward inflection point. The second downward inflection point (with further acceleration of the death rate) was noted at a median of 9.0 months after therapy initiation and 3.5 months after the first downward inflection point, with 59% of patients still alive at the time of the second inflection point. The OS half-life was a median of 20.4 months while patients were still on therapy vs. 7.3 months after completion of first-line chemotherapy.

Planning six rather than four cycles of first-line chemotherapy had a minimal impact on the PFS half-life (5.4 vs. 5.3 months, *p* = 0.57), while six planned cycles were associated with a non-significantly shorter OS than four cycles (9.5 vs. 10.4 months, *p* = 0.11).

### 3.5. PFS and Overall Survival Curve Characteristics for SCLC vs. Other Tumor Types

The population survival kinetics characteristics for extensive SCLC differed from those for other malignancies (Table 5). The PFS and OS curves were significantly (*p* < 0.0001) more likely to be moderately or highly convex than the curves we have previously assessed [9,10,12] for a broad range of other types of advanced solid tumors, including NSCLC; cancers of the breast, gastrointestinal tract, genitourinary tract, head and neck, etc.; sarcomas; and gliomas.

In other advanced malignancies, the PFS half-life correlated with OS half-life (*n* = 320, Spearman’s r = 0.81, *p* < 0.0001), but there was no significant correlation for extensive SCLC (*n* = 26, Spearman’s r = 0.22, *p* = 0.29). Similarly, post-progression survival (i.e., OS half-life minus PFS half-life) correlated significantly with PFS half-life for other malignancies (*n* = 376, Spearman’s r = 0.68, *p* < 0.0001) but not for extensive SCLC (*n* = 26, r = −0.33, *p* = 0.10).

### 3.6. PD-1/PD-L1 Inhibitors Combined with Chemotherapy

**Figure 2:**
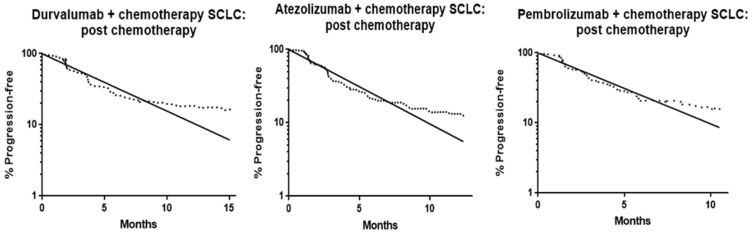
Log-linear plots of post-chemotherapy SCLC PFS curves for durvalumab, atezolizumab, and pembrolizumab, showing an inflection point to the right, in keeping with 2 distinct subpopulations with differing degrees of benefit from therapy. Dotted lines are Kaplan-Meier plots (on a log-linear scale), while the solid lines are log-linear regression lines.

All three PFS curves for chemotherapy combined with a PD-1/PD-L1 inhibitor were initially highly convex with rapid progression early after the completion of chemotherapy. In our landmark analyses of PD-1/PD-L1 inhibitor PFS curves after completion of chemotherapy, all three had a later inflection point to the right (Figure 2).

Longer follow up will be needed to determine if OS curves also display two-phase decay after chemotherapy completion.

The proportion of the post-chemotherapy population that belonged to the subpopulation with marked benefit (i.e., the proportion in the slow progression subpopulation) was 11% (95% confidence intervals 7–15%), 7% (3–22%,) and 8% (2–13%) for durvalumab, atezolizumab, and pembrolizumab, respectively. We multiplied these proportions by the proportion of the original population remaining progression-free at chemotherapy completion (88%, 89%, and 84%, respectively) to determine the proportion of the original population that appeared to benefit from immunotherapy (10%, 6%, and 7%, respectively).

The post-chemotherapy PFS half-lives for the rapidly progressing subpopulations were 2.9 (95% confidence intervals 2.5–3.3), 2.5 (2.2–2.8), and 2.5 (2.2–2.9) months, respectively, and the PFS half-lives for the slowly progressing subpopulations were 5 × 10^12^, 6 × 10^15^, and 2 × 10^12^ months, respectively. The 95% confidence intervals were too wide to be defined for the PFS half-lives in the slowly progressing subpopulations.

## 4. Discussion

Population survival kinetic analyses of PFS and OS curves can have several practical clinical implications. For example, they suggest the optimal frequency of follow-up scans for patients receiving systemic therapies [10], they make the PFS gain a better predictor of OS gain [9], they facilitate the estimation of the proportion of patients treated with curative intent who eventually relapse (and the half-life to relapse for those who relapse) [11,13], they identify the probable presence of two distinct subpopulations with differing rates of progression or death [12], and they enable the calculation of the proportion of patients with advanced disease who die per week that initiation of systemic therapy is delayed [15]. Our analyses provide added insight into SCLC behavior when treated with chemotherapy (alone or with PD-1/PD-1 inhibitors), give insight into the optimal frequency of follow-up scans during and after first-line chemotherapy, and drive home the need for better maintenance strategies after first-line chemotherapy.

Compared to other tumor types, extensive SCLC is much more likely to have PFS and OS curves that are highly or moderately convex on log-linear plots. This is in keeping with SCLC being very sensitive to chemotherapy but progressing rapidly when therapy is stopped after 4–6 cycles. There is an acceleration of tumor progression with an apparent downward inflection point in the PFS curves as soon as first-line chemotherapy is stopped and an acceleration of death rates with a downward inflection point in the OS curves a median of 1.7 months later.

As tumor growth accelerates, the proportion of remaining patients who would have progressed by the next scan increases substantially. This highlights the potential importance of conducting follow-up scans at relatively short intervals (e.g., every 3–6 weeks) after the cessation of first-line therapy if it is felt that the patient would be a reasonable candidate for second-line chemotherapy, when performance status is still good, and the tumor burden remains relatively small. With a longer delay prior to the initiation of second-line therapy, there is some possibility that efficacy would be greater due to the time-related reversal of some epigenetic-mediated resistance mechanisms [35] or due to the re-emergence of a sensitive tumor cell clone, but larger tumor burden and worsening performance status might make second-line therapy less effective if it is delayed. In addition, patients may have very rapid clinical deterioration as the cancer regrows, and the window of opportunity for retreatment may be missed if the detection of progression is late.

This late acceleration of progression and death is more likely due to the interruption of therapy than to the accelerated development of resistance to therapy. Several factors over time may lead to the development of acquired resistance to chemotherapy [35]. However, we would generally expect most of these to manifest themselves at a relatively constant rate, and it would be difficult to explain why the late acceleration of resistance would occur in SCLC to a greater extent than in other malignancies.

Our identification of two downward inflection points on the PFS and OS curves could have arisen from artefacts or from the overinterpretation of the data, but there could also have been a biological basis. Some patients with SCLC experience an improvement in symptoms as the tumor regresses but then an increase in symptoms as tumors begin to regrow just as the next treatment is due. These patients would be expected to progress rapidly when treatment is stopped, and we hypothesize that these could account for the first downward inflection point on the curve.

In these patients, if one restarted chemotherapy even shortly after the completion of first-line chemotherapy, the cancer burden could already be substantially larger than it was when first-line chemotherapy was completed. The resumption of chemotherapy might generally achieve little since the treatment was barely keeping ahead of the cancer while front-line chemotherapy was being administered. This could account for the group that is referred to as having “resistant relapse” SCLC [36].

However, cell exposure to chemotherapy can also be associated with an epigenetically mediated downregulation of membrane transporters, with reduced nutrient uptake into cells, reduced cell growth rate, and acquired resistance due to both cell quiescence and reduced chemotherapy uptake [35,37,38]. After initial tumor regression, the cancer would not be growing, but it would also not be substantially regressing further in response to chemotherapy. With increasing time after the last exposure to chemotherapy, this downregulation of the transport of nutrients and chemotherapy could reverse, with the resumption of tumor growth but also with the possibility of the return of at least some sensitivity to chemotherapy.

This can account for the group that is referred to as “sensitive relapse” SCLC [36]. Patients with SCLC with progression more than 3 months after therapy cessation are generally regarded as the sensitive relapse group [36]. This 3-month cutoff after chemotherapy completion is also the approximate time of the second PFS curve downward inflection point. We hypothesize that the second downward inflection point in the PFS curve might reflect the resumption of tumor cell growth (and partial chemosensitivity) in tumors that had become quiescent upon exposure to chemotherapy.

A variety of maintenance approaches have been tested. While some strategies have had no benefit, there has been a suggestion of benefit from other approaches. Examples of agents or maintenance strategies not having any benefit (although patient numbers and statistical power were low for some of these) include the TLR9 agonist lefitolimod [34], the monoclonal antibodies cixutumumab (targeting IGF1R) [17], tucotuzumab (targeting the epithelial cell adhesion molecule EpCAM) [39], rolitumumab (targeting hepatocyte growth factor) [32], or ganitumumab (targeting IGF1R) [32]; the monoclonal antibody-drug conjugate lorvotuzumab mertansine [27]; the angiogenesis inhibitors vandetanib [40,41] and thalidomide [42]; the Bcl-2 inhibitor obatoclax [33]; the hormone megesterol acetate [25]; the lipid-lowering agent pravastatin [26]; the anti-Hedgehog-pathway targeted agent vismodegib [17]; the immune checkpoint inhibitor ipilimumab [24,43]; and the topoisomerase I inhibitor irinotecan [44].

The multitargeted tyrosine kinase and angiogenesis inhibitor pazopanib significantly prolonged PFS from 1.8 months to 3.7 months but did not prolong OS [45]. The angiogenesis inhibitor bevacizumab increased the median PFS by approximately 1 month but with an inconsistent and statistically insignificant impact on OS [29,30]. The multitargeted tyrosine kinase inhibitor sunitinib also prolonged PFS by 1.6 months in a small, randomized phase II trial (*p* < 0.05), but the OS gain of 2.1 months was not statistically significant [46]. Interferon-α administration was associated with increased OS in one small study [47] and with a trend towards improved PFS and OS in another small study [48]. There was also a trend towards improved PFS and OS in a small study adding the PARP inhibitor veliparib to chemotherapy [22].

As noted, increasing the number of cycles of first-line chemotherapy was associated with an improvement in OS in some trials [3,4] but not others [5,6]. Maintenance therapy with the chemotherapy combination of cyclophosphamide, doxorubicin, and vincristine did not prolong OS in one small study [49] but was associated with prolonged complete response duration [50] and OS [4] in other extensive SCLC populations. In a trial accruing patients with both limited and extensive SCLC, maintenance with cyclophosphamide, methotrexate, and vincristine also significantly prolonged OS [51]. Maintenance topotecan prolonged PFS by 1.3 months but had no impact on OS [52].

Giving three cycles of maintenance oral etoposide after first-line chemotherapy improved PFS from 6.5 months to 8.5 months (*p* = 0.0018), with an improvement in OS from 11.2 to 12.2 months (*p* = 0.07) and an improvement in 3-year OS from 2% to 9% [53].

The experience with pemetrexed maintenance therapy in advanced NSCLC suggests a benefit of continuing maintenance therapy until tumor progression [54]. Prolonged maintenance oral etoposide might be well tolerated since etoposide causes relatively little cumulative toxicity. Maintenance oral etoposide (and other maintenance chemotherapy strategies) might be of little benefit for patients who have been experiencing rapid regrowth between cycles of front-line combination chemotherapy but could prolong the duration of time that quiescent tumors remain quiescent.

An early assessment of the administration of maintenance oral etoposide 100 mg/m^2^/day for 6 days every 3–4 weeks until tumor progression indicated that it was very well tolerated, with little cumulative toxicity and with the possible prolongation of PFS and OS [55]. In patients who were progression-free 3 weeks after the initiation of the last planned cycle of induction chemotherapy, the median post-induction PFS was 5.5 months in those receiving maintenance etoposide vs. 1.7 months in historical controls (*p* = 0.006).

The post-induction OS was 10.4 months in patients receiving maintenance oral etoposide vs. 6.8 months in historical controls (*p* = 0.04).

In patients who remained progression-free 3 weeks following the last induction cycle, the median PFS from the initiation of induction chemotherapy was 9.3 vs. 6.3 months with vs. without maintenance oral etoposide (*p* = 0.04) and the median OS from initiation of induction chemotherapy was 15.0 vs. 11.1 months (*p* = 0.047) [55]. Further assessment will be required to confirm the benefit of this approach.

PD-1/PD-L1 inhibitors also improve the outcome of extensive SCLC. Both durvalumab [8] and atezolizumab [7] were associated with a significant improvement in OS when added to chemotherapy, and pembrolizumab was associated with a significant improvement in PFS, but the improvement in OS did not achieve statistical significance [16].

Our post-chemotherapy landmark analyses suggest that the PFS gain with PD-1/PD-L1 inhibitors is primarily accounted for by marked benefit in 6–10% of the population, with little or no benefit in others. This subpopulation size is very similar to the proportion of patients with extensive SCLC that respond to single-agent PD-1/PD-L1 inhibitors [56,57,58,59,60].

The post-chemotherapy PFS half-lives of 2.5–2.9 months with the three PD-1/PD-L1 inhibitors is very similar to the median value for the post-chemotherapy PFS half-lives of 2.4 months for the trials without PD-1/PD-L1 inhibitors (derived from Table 2). Overall, the situation for SCLC appears to be very similar to that seen in other tumor types, where PD-1/PD-L1 inhibitors typically result in PFS curve two-phase decay, with one subpopulation deriving marked benefit and the other subpopulation appearing to derive little or no benefit [12]. This suggests that, for most patients, it would be unlikely that continuing PD-1/PD-L1 inhibitors after progression would add any benefit. On the other hand, patients with tumors sensitive to PD-1/PD-L1 inhibitors could continue to derive benefit from these agents long after chemotherapy is discontinued.

SCLC can be subdivided into four subtypes based on the expression of transcription factors [61]. The “inflamed” subtype accounts for about 17–18% of SCLCs, and patients with this subtype are more likely than others to derive benefit from PD-1/PD-L1 inhibitors [61]. The proportion of the SCLC population that derives benefit from PD-1/PD-L1 inhibitors (as assessed in our analyses or by single-agent response rates) is smaller than the size of the inflamed subtype population, and at least some patients from other SCLC subtypes appear to derive benefit from these agents [61]. This suggests that the inflamed subtype is enriched in factors required for therapy benefit, but not all patients with the inflamed subtype have the required sensitivity factors, and a small proportion of patients from other subtypes may also possess them.

As in other analyses we published [13], the estimated PFS half-life for the subpopulation deriving marked benefit from PD-1/PD-L1 inhibitors was probably higher than the true PFS half-life for this subpopulation, and the 95% confidence intervals were too wide to permit their calculation. Much longer follow-up will be needed to more accurately define the PFS half-life for the good-outcome subpopulation.

In light of the potential benefit of maintenance oral etoposide in extensive SCLC, it would be reasonable to assess whether adding maintenance oral etoposide to a maintenance PD-1/PD-L1 inhibitor would be of value.

Our analysis had several limitations. We made the assumption that PFS and OS curves typically follow first-order kinetics and that deviations of log-linear plots of these curves reflect underlying biological or treatment properties, in keeping with our earlier assessments of a range of therapies and situations [9,10,11,12,13,15].

Our assigning of a specific curve shape classification to a given log-linear curve was subjective. Many curves could be included in either of two different shape classifications (e.g., either one-phase vs. variable one-phase, one-phase vs. low convexity or moderate convexity vs. high convexity). Shifting the PFS and OS curves for individual studies from one appropriate classification to another appropriate classification had no impact on any of our conclusions.

As noted previously, our identification of two downward inflection points on the PFS and OS curves could have arisen from artefacts or from the overinterpretation of the data.

We could have introduced bias by excluding trials with fewer than 50 patients, trials published after our initial data cutoff date of November 2019, or trials that included other chemotherapy agents. Furthermore, we did not specifically record data on trials that we excluded from our analysis. These types of analyses can also be impacted by publication bias since publication is more likely for positive trials than for negative trials, and this can impact the generalizability of the results.

There could also have been bias in choosing the truncation point for curves in calculating the PFS and OS half-lives (i.e., the point estimated to have fewer than 10 remaining patients).

The trials to which we compared SCLC trials involved a heterogeneous range of tumor and therapy types.

We suggest conducting more frequent follow-up scans after completion of first line therapy, but the cost-effectiveness of this would need to be assessed.

## 5. Conclusions

Overall, our assessment of population survival kinetics of platinum–etoposide chemotherapy in extensive SCLC highlights the rapid tumor progression that occurs after the completion of first-line chemotherapy and suggests that frequent follow-up scans and new maintenance strategies should be explored. Our analyses suggest that only a small subpopulation of patients with SCLC derive benefit from PD-1/PD-L1 inhibitors, and they derive marked benefit, with little apparent benefit in the majority.

## Figures and Tables

**Table 1:** Extensive small-cell lung cancer studies included platinum and etoposide alone or with a targeted agent.

Author	Platinum	Targeted Agent	No. Patients	No. Cycles	Median PFS (Months)	Median OS (Months)
Belani [17]	cisplatin	vismodegib	52	4	4.4	9.8
Belani [17]	cisplatin	cixutumumab	52	4	4.6	10.1
Glisson [32]	cis- or carboplatin		61	6	5.4	10.8
Glisson [32]	cis- or carboplatin	ganitumumab	62	6	5.5	10.7
Glisson [32]	cis- or carboplatin	rolitumumab	62	6	5.4	12.2
Horn [7]	carboplatin	atezolizumab	201	4	5.2	12.3
Horn [18]	cisplatin	bevacizumab	63	4	4.7	10.9
Horn [7]	carboplatin		202	4	4.3	10.3
Jalal [19]	carboplatin		94	4–6	Not reported	10.4
Kim [20]	cisplatin		189	6	5.8	10.3
Langer [33]	carboplatin		82	6	5.2	9.8
Langer [33]	carboplatin	obatoclax	83	6	5.8	10.5
Niell [21]	cisplatin		282	6	5.9	9.9
Owonikoko [22]	cisplatin	veliparib	52	4	6.1	10.3
Paz-Ares [8]	cis- or carboplatin		269	4	5.4	10.3
Paz-Ares [8]	cis- or carboplatin	durvalumab	268	4	5.1	13
Pirker [23]	cis- or carboplatin	darbepoetin	298	6	5.5 ^a^	9.2
Pirker [23]	cis- or carboplatin		298	6	5.5 ^a^	9.2
Reck [24]	cis- or carboplatin		476	4	4.4	10.9
Reck [24]	cis- or carboplatin	ipilimumab	478	4	4.6	11
Rowland [25]	cisplatin		121	4	7.2	10
Rowland [25]	cisplatin	megesterol	122	4	6.1	8.2
Seckl [26]	cis- or carboplatin	pravastatin	239	6	Not reported	9.1
Seckl [26]	cis- or carboplatin		243	6	Not reported	8.8
Socinski [27]	carboplatin	lorvotuzumab mertansine	94	6	6.2	10.1
Socinski [28]	carboplatin		455	6	5.4	10.6
Spigel [29]	cis- or carboplatin		50	4	4.4	10.9
Thomas [34]	cis- or carboplatin	lefitolimod	62	4	3.0	9.2
Spigel [29]	cis- or carboplatin	bevacizumab	52	4	5.5	9.4
Tiseo [30]	cisplatin	bevacizumab	101	6	6.7	9.8
Tiseo [30]	cisplatin		103	6	5.7	8.9
Viren [31]	carboplatin		56	6	4.6 ^a^	4.6
Median					5.4	10.2

^a^ Median PFS reported, but PFS half-life could not be calculated since there were no published PFS curves.

**Table 2:** PFS curve characteristics and half-lives.

Author ^a^	No. Cycles	PFS Curve Shape ^b^	PFS t_1/2_^d^	LCI ^e^	UCI ^f^	R^2 g^	1st Inflec ^h^	1st Inflec from End Rx ^i^	1st Inflec % Left ^j^	2nd Inflec ^k^	2nd Inflec % Left ^l^	PFS t_1/2_ on Rx ^m^	PFS t_1/2_ Post Rx ^n^	PFS t_1/2_ Post 2nd Inflec ^o^
Belani (vism) [17]	4	high conv	5.0	4.4	5.7	0.74	3.0	0.2	81%	4.2	64%	11.0	2.0	1.0
Belani (cixut) [17]	4	high conv	5.4	4.5	6.5	0.59	2.7	−0.1	93%	4.0	83%	37.7	2.2	1.0
Glisson [32]	6	high conv	5.2	4.7	5.6	0.84	3.6	−0.6	80	5.4	55%	11.9	2.1	1.7
Glisson (ganit) [32]	6	high conv	5.4	5.0	5.9	0.82	2.7	−1.5	85	5.5	52%	9.3	2.0	1.5
Glisson (rolit) [32]	6	high conv	5.7	5.2	6.3	0.78	3.8	−0.4	81	5.4	49%	12.4	1.9	1.7
Horn (atezo) [7]	4	high conv ^c^	4.9	4.6	5.2	0.88	2.7	−0.1	86%	4.0	76%	16.1	3.0	2.4
Horn (bev) [18]	4	high conv	4.9	4.3	5.7	0.82	3.8	1.0	75%	3.8	75%	8.0	2.0	1.5
Horn [7]	4	high conv	4.1	3.7	4.5	0.83	2.7	−0.1	91%	3.8	75%	17.1	2.0	1.4
Jalal [19]	4–6													
Kim [20]	6	mod conv	4.7	4.5	5.0	0.93	4.1	−0.1	66%	7.0	32%	6.6	2.6	1.8
Langer [33]	6	mod conv	4.1	3.9	4.4	0.91	3.8	−0.4	64%	5.3	49%	5.5	2.0	1.4
Langer (obat) [33]	6	high conv	4.9	4.4	5.3	0.84	5.0	0.8	67%	6.8	33%	10.4	2.1	1.6
Niell [21]	6	mod conv	5.2	4.8	5.6	0.91	4.6	0.4	68%	6.4	46%	9.6	2.6	2.1
Owonikoko (vel) [22]	4	high conv	6.6	5.7	7.7	0.69	3.0	0.2	92%	5.7	67%	27.9	3.4	1.1
Paz-Ares [8]	4	high conv	5.3	4.8	5.8	0.82	4.6	1.8	73%	6.3	44%	13.5	3.1	1.7
Paz-Ares (durv) [8]	4	high conv ^c^	5.6	5.3	6.1	0.90	4.5	1.7	73%	6.7	41%	15.0	3.7	7.3
Pirker (darb) [23]	6													
Pirker [23]	6													
Reck [24]	4	high conv ^c^	4.4	3.9	4.9	0.77	2.8	0.0	92%	4.0	77%	84.5	1.9	1.1
Reck (ipil) [24]	4	high conv ^c^	4.6	4.2	5.1	0.83	2.8	0.0	94%	3.9	77%	79.7	2.2	1.4
Rowland [25]	4	high conv	6.4	5.8	7.0	0.88	2.8	0.0	88%	8.9	37%	19.2	4.4	2.0
Rowland (meg) [25]	4	mod conv	5.9	5.6	6.2	0.96	5.3	2.5	60%	8.2	37%	7.2	4.6	3.2
Secki (prav) [26]	6													
Secki [26]	6													
Socinski (lorv) [27]	6	high conv ^c^	6.5	5.9	7.1	0.84	3.2	−1.0	87%	4.5	73%	16.2	3.0	3.0
Socinski [28]	6	high conv	6.0	5.5	6.6	0.87	1.4	−2.8	93%	4.5	73%	11.8	2.9	2.7
Spigel [29]	4	high conv	5.5	4.7	6.4	0.78	1.3	−1.5	90%	3.9	71%	9.9	2.5	1.0
Spigel (bev) [29]	4	mod conv	6.5	5.9	7.1	0.84	3.9	1.1	77%	5.4	58%	9.6	3.3	1.9
Thomas (lefi) [34]	4	high conv	3.1	2.7	3.6	0.84	1.6	−1.2	87%	3.6	44%	4.4	1.8	1.2
Tiseo (bev) [30]	6	high conv	6.7	6.0	7.5	0.89	4.4	0.2	76%	5.9	62%	12.0	3.6	3.0
Tiseo [30]	6	high conv	5.5	4.9	6.3	0.89	2.7	−1.5	82%	4.6	70%	9.4	2.6	2.5
Viren [31]	6													
Medians			5.4	4.8	5.9	0.84	3.1	0.0	82%	5.4	60%	11.9	2.6	1.7

^a^ Agents administered with platinum–etoposide: vism: vismodegib; cixut: cixutumumab; ganit: ganitumumab; rolit: rolitumumab; atezo: atezolizumab; bev: bevacizumab; obat: obatoclax; vel: veliparib; durv: durvalumab; darb: darbepoetin; ipil: ipilimumab; meg: megesterol acetate; prav: pravastatin; lorv: lorvotuzumab mertansine; lefi: lefitolimod; ^b^ high conv: highly convex; mod conv: moderately convex; ^c^ convex but with rightward deviation in terminal portion; ^d^ PFS t_1/2_: PFS half-life (months); ^e^ LCI: lower limit for 95% confidence interval for PFS t_1/2_; ^f^ UCI: upper limit for 95% confidence interval for PFS t_1/2_; ^g^ R^2^ value for fit of 1-phase exponential decay model to PFS data; ^h^ time (months) from initiation of therapy to first downward inflection point in PFS curve; ^i^ time (months) of first downward inflection point minus the end of the last planned chemotherapy cycle (i.e., from 3 weeks beyond day 1 of the final cycle); ^j^ % of population still progression-free at time of onset of first downward inflection point in PFS curve; ^k^ time (months) from initiation of therapy to second downward inflection point in PFS curve; ^l^ % of population still progression-free at time of onset of second downward inflection point in PFS curve; ^m^ PFS half-life (months) up to end of last planned cycle (4th or 6th) of chemotherapy (i.e., to 3 weeks beyond day 1 of the final cycle); ^n^ PFS half-life (months) after end of last planned cycle (4th or 6th) of chemotherapy (i.e., from 3 weeks beyond day 1 of the final cycle); ^o^ PFS half-life (months) after onset of second downward inflection point in PFS curve.

**Table 3:** Proportion of remaining patients progressing by the next scan for scans performed at different intervals.

	Interval (Weeks) Between Follow-Up Scans				
	3 weeks	6 weeks	9 weeks	12 weeks	18 weeks
	Proportion of remaining patients progressing since previous scan				
During 1st-line therapy (PFS t_1/2_ 11.9 months/ 51.6 weeks)	4%	8%	11%	15%	21%
After 1st-line therapy completion (PFS t_1/2_ 2.6 months/11.3 weeks)	17%	31%	42%	51%	67%
After 2nd PFS curve inflection point (PFS t_1/2_ 1.7 months/7.4 weeks)	24%	43%	57%	67%	81%

**Table 4:** OS curve characteristics and half-lives.

Author ^a^	No. Cycles	OS Curve Shape ^b^	OS t_1/2_^d^	LCI ^e^	UCI ^f^	R^2 g^	PPS ^h^	1st Inflec ^i^	1st Inflec from End Rx ^j^	1st Inflec % Left ^k^	2nd Inflec ^l^	2nd Inflec % Left ^m^	OS t_1/2_ on Rx ^n^	OS t_1/2_ Post Rx ^o^
Belani (vism) [17]	4	high conv	9.9	9.3	10.5	0.92	4.9	2.9	0.1	96%	8.2	66%	33.7	7.3
Belani (cixut) [17]	4	high conv	10.4	9.5	11.4	0.83	5.0	5.3	2.5	88%	11.8	42%	53.6	7.5
Glisson [32]	6	high conv	10.2	9.4	11.1	0.82	5.0	3.6	−0.6	93%	9.0	68%	27.9	8.2
Glisson (ganit) [32]	6	mod conv	10.1	9.6	10.6	0.93	4.7	7.1	2.9	73%	13.6	39%	16.5	7.3
Glisson (rolit) [32]	6	mod conv	11.0	10.6	11.5	0.93	5.3	5.7	1.5	82%	13.1	45%	25.0	8.4
Horn (atezo) [7]	4	mod conv	14.4	13.7	15.1	0.90	9.5	5.3	2.5	90%	10.2	61%	39.9	11.0
Horn (bev) [18]	4	high conv	10.4	9.3	11.5	0.87	5.5	5.5	2.7	83%	11.5	86%	25.3	7.8
Horn [7]	4	high conv	10.7	10.0	11.4	0.86	6.6	4.3	1.5	91%	6.5	79%	26.8	8.0
Jalal [19]	4–6	high conv	10.9	9.8	12.2	0.89		5.0	0.8	89%	12.8	44%	36.2	7.1
Kim [20]	6	mod conv	9.5	8.9	10.1	0.91	4.8	6.6	2.4	74%	11.5	43%	17.7	6.7
Langer [33]	6	mod conv	8.6	8.4	8.9	0.96	4.5	5.7	1.5	77%	10.0	47%	9.2	7.1
Langer (obat) [33]	6	high conv	10.7	10.4	11.2	0.94	5.8	6.2	2.0	79%	15.8	34%	17.8	8.3
Niell [21]	6	mod conv	9.5	9.0	10.1	0.93	4.3	6.1	1.9	78%	12.8	35%	21.0	6.5
Owonikoko (vel) [22]	4	high conv	11.9	10.4	13.6	0.74	5.3	5.6	2.8	90%	7.8	76%	85.6	7.4
Paz-Ares [8]	4	mod conv	10.0	9.5	10.6	0.94	4.7	4.3	1.5	86%	6.3	75%	18.0	7.9
Paz-Ares (durv) [8]	4	mod conv	12.8	12.3	13.3	0.96	7.2	5.8	3.0	80%	14.1	49%	19.7	11.2
Pirker (darb) [23]	6	mod conv ^c^	8.2	7.8	8.5	0.97		6.4	2.2	66%	13.0	26%	10.8	6.4
Pirker [23]	6	mod conv	8.1	7.7	8.4	0.96		5.0	0.8	78%	7.7	57%	10.7	5.9
Reck [24]	4	mod conv	9.9	9.1	10.7	0.85	5.5	5.5	2.7	89%	8.8	65%	233.5	7.3
Reck (ipil) [24]	4	mod conv ^c^	10.3	9.7	10.9	0.89	5.7	6.3	3.5	84%	9.6	59%	154.7	7.6
Rowland [25]	4	mod conv	8.3	7.7	9.0	0.89	1.9	4.9	2.1	81%	9.1	56%	16.9	6.7
Rowland (meg) [25]	4	mod conv	7.0	6.7	7.3	0.97	1.1	6.3	3.5	61%	8.4	47%	6.3	6.4
Secki (prav) [26]	6	mod conv	8.1	7.4	8.9	0.94		5.8	1.6	74%	7.8	60%	13.7	5.5
Secki [26]	6	high conv	9.6	8.6	10.7	0.86		4.6	0.4	84%	6.6	70%	16.8	5.4
Socinski (lorv) [27]	6	high conv ^c^	12.9	11.9	14.1	0.85	6.4	5.2	1.0	88%	7.6	74%	43.9	7.9
Socinski [28]	6	high conv ^c^	11.1	10.4	11.9	0.89	5.1	5.1	0.9	83%	10.0	53%	21.2	7.3
Spigel [29]	4	mod conv	13.9	12.6	15.5	0.88	8.4	3.7	0.9	92%	6.3	79%	36.2	9.6
Spigel (bev) [29]	4	high conv ^c^	11.0	10.0	12.1	0.84	4.5	3.4	0.6	88%	8.7	63%	14.1	8.3
Thomas (lefi) [34]	4	high conv	9.1	8.4	9.9	0.89	6.0	5.6	2.8	79%	9.0	54%	30.9	6.6
Tiseo (bev) [30]	6	mod conv ^c^	9.5	9.0	10.1	0.94	2.8	6.0	1.8	75%	8.9	59%	15.2	7.1
Tiseo [30]	6	high conv	8.3	7.6	9.1	0.91	2.8	5.7	1.5	74%	7.6	65%	12.4	5.6
Viren [31]	6	high conv	5.0	4.7	5.5	0.77		3.5	−0.7	74%	4.5	60%	7.3	1.0
Medians			10.1	9.3	10.7	0.90	5.1	5.5	1.7	83%	9.0	59%	20.4	7.3

^a^ Agents administered with platinum–etoposide: vism: vismodegib; cixut: cixutumumab; ganit: ganitumumab; rolit: rolitumumab; atezo: atezolizumab; bev: bevacizumab; obat: obatoclax; vel: veliparib; durv: durvalumab; darb: darbepoetin; ipil: ipilimumab; meg: megesterol acetate; prav: pravastatin; lorv: lorvotuzumab mertansine; lefi: lefitolimod; ^b^ high conv: highly convex; mod conv: moderately convex; ^c^ convex, but with rightward deviation in terminal portion; ^d^ OS t_1/2_: OS half-life (months); ^e^ LCI: lower limit for 95% confidence interval for OS t_1/2_; ^f^ UCI: upper limit for 95% confidence interval for OS t_1/2_; ^g^ R^2^ value for fit of 1-phase exponential decay model to OS data; ^h^ PPS: post-progression survival: OS half-life minus PFS half-life; ^i^ time (months) from initiation of therapy to first downward inflection point in OS curve; ^j^ time (months) of first downward inflection point minus the end of the last planned chemotherapy cycle (i.e., minus 3 weeks beyond day 1 of the final cycle); ^k^ % of population still alive at time of onset of first downward inflection point in OS curve; ^l^ time (months) from initiation of therapy to second downward inflection point in OS curve; ^m^ % of population still alive at time of onset of second downward inflection point in OS curve; ^n^ OS half-life (months) up to end of last planned cycle (4th or 6th) of chemotherapy; ^o^ OS half-life (months) after end of last planned cycle (4th or 6th) of chemotherapy.

**Table 5:** Moderately and highly convex PFS and OS curves in extensive SCLC vs. other tumor types.

	Extensive SCLC Treated with Platinum–Etoposide	Other Advanced Cancers/Therapy Types [9,10,12]	*p* (SCLC vs. Others) ^a^
Total No. evaluable PFS curves	26	888	
No. (%) PFS curves highly convex	21 (81%)	35 (4%)	<0.0001
No. (%) PFS curves highly or moderately convex	26 (100%)	186 (21%)	<0.0001
Total No. evaluable OS curves	32	363	
No. (%) OS curves highly convex	15 (47%)	15 (4%)	<0.0001
No. (%) OS curves highly or moderately convex	32 (100%)	87 (24%)	<0.0001

^a^ Fisher’s exact test.

## Data Availability

All data we assessed are readily available since they were derived from the published literature. The calculations we performed on the data are included in the tables.
